# A Practical Treatise on Fractures and Dislocations

**Published:** 1910-07

**Authors:** 


**A Practical Treatise on Fractures and Dislocations.** By **Lewis A. Stimson,** B.A., M.D., Professor of Surgery in Cornell University Medical College, New York. Sixth edition. Illustrated. Octavo, pp. 876. New York and Philadelphia: Lea & Febiger. (Cloth, $5.00.)

This author sends forth another revision of his book. All of the good qualities of the five previous editions have been retained and such new additions as a closer study of the subject, aided and stimulated by *x*-ray examinations warrants. Stimson has had an enormous experience in his chosen field, and his treatise now takes rank as a leading authority on fractures and dislocations, being accepted as such in the courts of law. Everything that was said in praise of former revisions applies to this one which is a credit to author and publishers. J. A. R.

